# Surgery and Suicide Deaths Among Patients With Cancer

**DOI:** 10.1001/jamanetworkopen.2024.31414

**Published:** 2024-09-03

**Authors:** Michael L. Chen, Scarlett L. Gomez, Ruth O’Hara, Esther M. John, Arden M. Morris, Allison W. Kurian, Eleni Linos

**Affiliations:** 1 Center for Digital Health, Stanford University, Stanford, California; 2 Department of Dermatology, Stanford University School of Medicine, Stanford, California; 3 Department of Epidemiology and Biostatistics, University of California, San Francisco; 4 Department of Psychiatry and Behavioral Sciences, Stanford University, Stanford, California; 5 Sierra Pacific Mental Illness, Research, Education, and Clinical Center, Veterans Affairs Palo Alto Health Care System, Palo Alto, California; 6 Department of Epidemiology and Population Health, Stanford University School of Medicine, Stanford, California; 7 Department of Medicine, Stanford University School of Medicine, Stanford, California; 8 Stanford Cancer Institute, Stanford University School of Medicine, Stanford, California; 9 Department of Surgery, Stanford University School of Medicine, Stanford, California; 10 Stanford-Surgery Policy Improvement Research and Education Center, Stanford University, Stanford, California

## Abstract

This cohort study describes suicide incidence among patients with cancer compared with the general population and by surgical status and cancer treatment.

## Introduction

Suicide is a critical public health issue with a higher incidence in patients who underwent cancer surgery.^1^ However, the association of undergoing different cancer treatments with suicide risk are not well understood. We investigated whether specific cancer treatment modalities were associated with suicide deaths.

## Methods

We queried the Surveillance, Epidemiology, and End Results (SEER) Program 17 Registries database (2000-2020) for patients with a first primary diagnosis of the 15 most common solid-organ cancers (SEER recode *International Classification of Diseases for Oncology, Third Revision* codes). The Stanford Institutional Review Board deemed this study exempt from review and waived the informed consent requirement because public, deidentified data were used. We followed the STROBE reporting guideline.

We identified each patient’s surgical status (surgery performed, yes or no), surgical recommendation (yes or no), radiotherapy (yes or no), and chemotherapy (yes or no) for first-course treatment. Surgical recommendation defaults to not recommended and is adjusted by independent registries to recommended pending appropriate case-by-case evidence; therefore, the recommended surgical subgroup has high precision but is not necessarily complete (eMethods 1 in Supplement 1). Standardized mortality ratios (SMRs) based on suicide deaths were calculated using SEER*Stat (eMethods 2 in Supplement 1).^2^ Two-sided *P* < .05 indicated statistical significance. Data analysis was performed September 2023 to July 2024.

## Results

Among 5 164 987 patients (2 518 775 females [48.8%], 2 646 212 males [51.2%]; median [IQR] age at diagnosis, 62 [52-72] years) with a first primary cancer diagnosis, 7132 (0.1%) died by suicide. Mean person-years at risk was 5.87 years.

**Figure 1.**
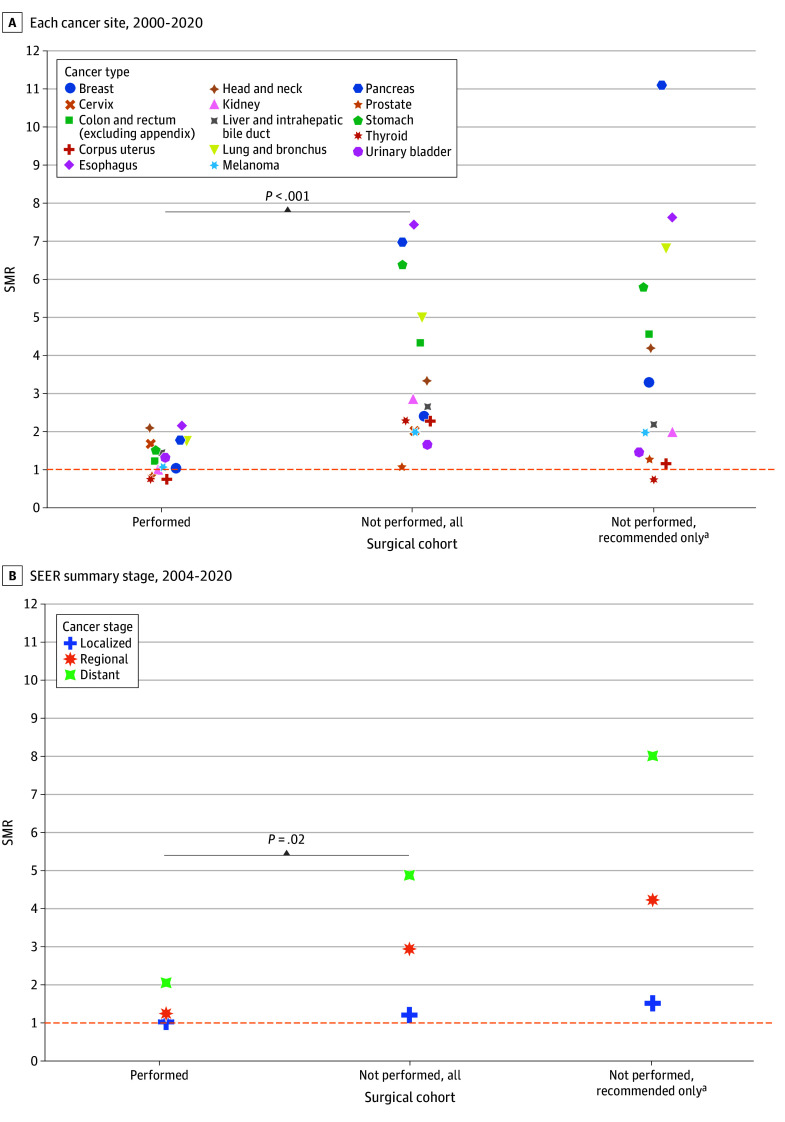
Standardized Mortality Ratio (SMR) of Suicide by Surgical Therapy Status and Recommendation *P* values show the difference in SMRs between patients who underwent surgery vs patients who did not undergo surgery and adjusted for cancer type and cancer stage. SEER indicates Surveillance, Epidemiology, and End Results program. ^a^This cohort comprises patients for whom there is a high reliability that surgery was recommended (described in eMethods 1 in Supplement 1), and it is a subgroup of the not performed, all cohort.

**Figure 2.**
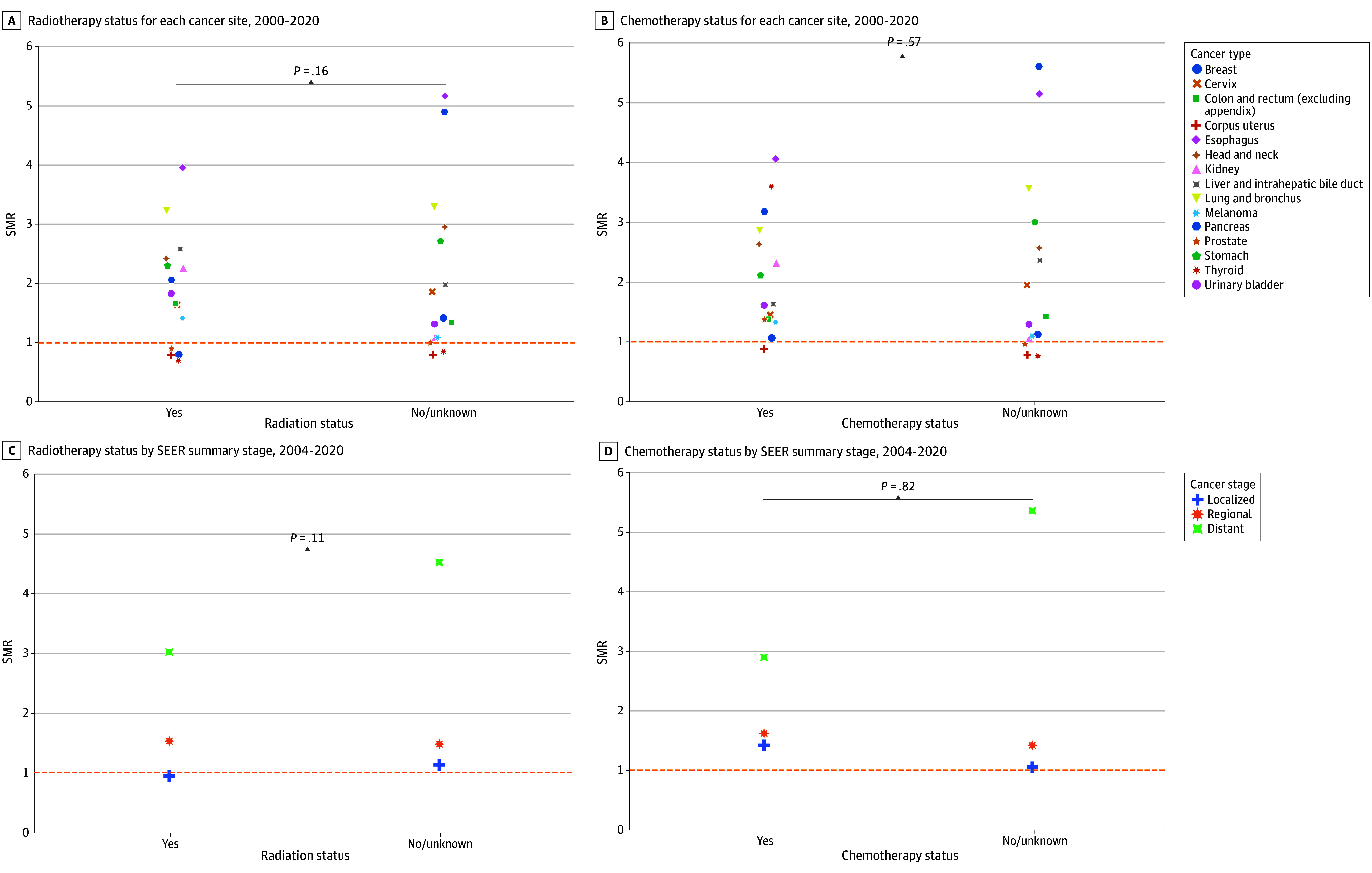
Standardized Mortality Ratio (SMR) of Suicide by Radiotherapy and Chemotherapy Status for Each Cancer Site and Stage *P* values show the difference in SMRs by radiotherapy status adjusted for cancer type, chemotherapy status adjusted for cancer type, chemotherapy status adjusted for cancer stage, and chemotherapy status adjusted for cancer stage. SEER indicates Surveillance, Epidemiology, and End Results program.

For patients who did not undergo surgery, suicide deaths were highest for pancreatic, esophageal, lung or bronchial, and stomach cancers (Figure 1A). SMRs varied by surgical status, with increased suicide deaths among patients who did not undergo surgery (*P* < .001, meta-regression of logarithmic SMRs). Among patients who did not undergo surgery, those who were recommended surgery with high reliability had the highest suicide deaths across all cancer stages (Figure 1B). For example, patients with pancreatic cancer had 331% higher suicide incidence than the general population overall, but this rate varied by treatment: 77% higher with surgery, 598% higher without surgery, and 1011% higher without surgery even though surgery was recommended. Subgroup analyses by cancer stage, race and ethnicity, age, and sex by surgical status illustrated similar patterns. Undergoing radiotherapy or chemotherapy was not associated with suicide deaths (Figure 2).

## Discussion

We identified differences in the excess incidence of suicide by treatment received. Increased suicide deaths among patients who did not undergo surgery may be partly due to these patients having more advanced or inoperable tumors. However, even among patients with advanced cancer stage, suicide deaths were lower among those who underwent surgery, suggesting that advanced stage alone does not account for differences by treatment. Other factors that may play a role in differences in suicide deaths by surgical status include factors that were not captured by staging, medical contraindications, and social or psychological comorbidities.^3^ Surgery can provide patients hope for an improved quality of life^4^; one hypothesis is that surgery may give patients a sense of hope, thus preventing suicide.

Higher suicide deaths among patients who did not undergo recommended surgery has multiple possible explanations. Refusal of surgery may be an early sign of higher suicide risk. Patient-elected death may reflect their loss of autonomy, dignity, or functional status.^5^ Varying access to cancer surgery may increase disparities in access to recommended surgery.^6^

Study limitations include SEER capturing approximately half of US patients with cancer, limitation to first-course treatment, lack of information on psychiatric conditions, and inability to ascertain patient motivators to pursue or decline therapy. These findings warrant further investigation into reasons for higher suicide deaths among patients who did not undergo surgery, including the role played by comorbid psychiatric conditions, lack of hope for the future, quality-of-life changes, and surgical access barriers.

## Notes

Supplement 1. **eMethods 1.** SEER Definition for Recommendation for Surgery **eMethods 2.** Statistical Methods

Supplement 2.
Data Sharing Statement
