# Supplementary material for: Surgery and Suicide Deaths Among Patients With Cancer
Source: JAMA Netw Open. 2024 Sep 3;7(9):e2431414. doi: 10.1001/jamanetworkopen.2024.31414 (PMC11372498; doi:10.1001/jamanetworkopen.2024.31414)
Supplement: Supplement 1. — eMethods 1. SEER Definition for Recommendation for Surgery eMethods 2. Statistical Methods [file jamanetwopen-e2431414-s001.pdf]

Supplemental Online Content

Chen ML, Gomez SLL, O'Hara R, et al. Surgery and suicide deaths among patients with cancer. *JAMA Netw Open*. 2024;7(9):e2431414.
doi:10.1001/jamanetworkopen.2024.31414

eMethods 1. SEER Definition for Recommendation for Surgery

eMethods 2. Statistical Methods

This supplemental material has been provided by the authors to give readers additional information about their work.

© 2024 Chen ML et al. *JAMA Network Open*

eMethods 1. SEER Definition for Recommendation for Surgery

SEER defines a recommendation for surgery as (1) an admission or documentation of surgery, or (2) a referral to a surgeon in the absence of either known contraindications or an offered alternative treatment modality. When surgical status is coded as “not performed,” most SEER registry classification systems default a patient’s surgical recommendation status to a “not recommended” code. Each reporting facility independently reviews surgical recommendation statuses and, pending appropriate evidence, adjusts the surgical recommendation statuses to “recommended” when deemed appropriate. As such, the SEER code of “recommended” for surgery is generally reliable, whereas the SEER code for “not recommended” for surgery may contain misclassifications. In the current study, we define only patients with the higher reliability SEER code of “recommended” for surgery who did not undergo surgery within the “Not performed, recommended only” surgical subgroup.

eMethods 2. Statistical Methods

We computed standardized mortality ratios (SMRs) as the number of suicide deaths among cancer cases (by treatment category) relative to the expected suicide mortality in the U.S. general population based on U.S. mortality data from the National Center for Health Statistics, as calculated within SEER*Stat. Suicide death and other causes of death were based on ICD-10 codes as available in the cause of death recode variable in SEER. Patients who died of non-suicide-related death or have not died were categorized in the “Did Not Die By Suicide” group. The follow-up period for patients was censored on the date of death for patients who died of non-suicide-related death. The calculation of SMRs was previously described in Andersen et al. (2014). Using SEER*Stat, SMRs were adjusted to the general population for age (5-year intervals through 84 years and one group for 85+ years), sex (male, female), race (White/other unspecified, Black, Other [American Indian, Alaskan Native, Asian/Pacific Islander]), and calendar-year of death and stratified for each cancer type. Follow-up for vital status was from date of diagnosis (starting in January 2000) through December 2020 (or, if deceased, date of death). The combined SEER summary stage, defined as *in situ*, localized, regional, or distant, was identified for each patient diagnosed from 2004-20 and used to stratify SMRs. Stratification of SMRs by race/ethnicity, age, and sex was also reported in the Supplementary Materials. We followed the Strengthening the Reporting of Observational Studies in Epidemiology (STROBE) reporting guidelines. Statistical testing was conducted via meta-regression of logarithmic SMRs by therapy status and adjusted for the covariates of cancer type or stage (SEER*Stat 8.4.2 and Python 3.8). A significance level of 0.05 was used, and two-sided hypothesis testing was reported.

© 2024 Chen ML et al. *JAMA Network Open*
