# Supplementary material for: Surgery and Suicide Deaths Among Patients With Cancer
Source: JAMA Netw Open. 2024 Sep 3;7(9):e2431414. doi: 10.1001/jamanetworkopen.2024.31414 (PMC11372498; doi:10.1001/jamanetworkopen.2024.31414)
Supplement: Supplement 2. — Data Sharing Statement [file jamanetwopen-e2431414-s002.pdf]

Data Sharing Statement

Chen. Surgery and Suicide Deaths Among Patients With Cancer. *JAMA Netw Open*. Published September 03, 2024. doi:10.1001/jamanetworkopen.2024.31414

Data
Data available: No

Additional Information
Explanation for why data not available: Data are publicly available via the Surveillance, Epidemiology, and End Results Program after completion of the Access Request Form.
